# Vervet monkeys use paths consistent with context‐specific spatial movement heuristics

**DOI:** 10.1002/ece3.1755

**Published:** 2015-10-05

**Authors:** Julie A. Teichroeb

**Affiliations:** ^1^ Department of AnthropologyUniversity of Toronto Scarborough1265 Military TrailTorontoOntarioM1C 1A4Canada

**Keywords:** Cercopithecines, decision‐making, navigation, optimal Hamiltonian path problem, primates, rules of thumb, traveling salesman problem

## Abstract

Animal foraging routes are analogous to the computationally demanding “traveling salesman problem” (TSP), where individuals must find the shortest path among several locations before returning to the start. Humans approximate solutions to TSPs using simple heuristics or “rules of thumb,” but our knowledge of how other animals solve multidestination routing problems is incomplete. Most nonhuman primate species have shown limited ability to route plan. However, captive vervets were shown to solve a TSP for six sites. These results were consistent with either planning three steps ahead or a risk‐avoidance strategy. I investigated how wild vervet monkeys (*Chlorocebus pygerythrus*) solved a path problem with six, equally rewarding food sites; where site arrangement allowed assessment of whether vervets found the shortest route and/or used paths consistent with one of three simple heuristics to navigate. Single vervets took the shortest possible path in fewer than half of the trials, usually in ways consistent with the most efficient heuristic (the convex hull). When in competition, vervets' paths were consistent with different, more efficient heuristics dependent on their dominance rank (a cluster strategy for dominants and the nearest neighbor rule for subordinates). These results suggest that, like humans, vervets may solve multidestination routing problems by applying simple, adaptive, context‐specific “rules of thumb.” The heuristics that were consistent with vervet paths in this study are the same as some of those asserted to be used by humans. These spatial movement strategies may have common evolutionary roots and be part of a universal mental navigational toolkit. Alternatively, they may have emerged through convergent evolution as the optimal way to solve multidestination routing problems.

## Introduction

The “traveling salesman (or salesperson) problem” (TSP) is a classic optimal foraging problem where an individual must choose the shortest route through multiple sites before returning to the starting location (Lawler et al. 1985). For foraging animals visiting several food sources, solving the TSP would be adaptive, leading to maximum energy gains with the least energy output (Pyke 1984). However, the TSP is a combinatorial optimization problem, part of a class of mathematical problems termed NP hard (NP = nondeterministic polynomial). Solutions to TSPs become increasingly complex and intractable as the number of destinations increases (Lawler et al. 1985; MacGregor and Chu 2011). For example, with six targets, an individual can take 60 possible routes ((*n*−1)!/2, where *n* is the number of targets), and by nine targets, this number jumps to 20,160.

Most research examining how animals approximate optimal solutions to TSP‐like problems has focused on humans. Rather than calculating all possible routes, humans generate fairly accurate solutions using simple heuristics (MacGregor and Chu 2011). Cognitive heuristics are fast, frugal, adaptive “rules of thumb” that can be applied to a situation to arrive at a reasonable course of action (Gigerenzer and Todd 1999). Examples of heuristics used by humans to solve the TSP are the nearest neighbor rule (choosing the closest site that has not been visited) and the least‐decision‐load strategy (choosing the route with the fewest movement decisions) (Wiener et al. 2006; MacGregor and Chu 2011). Although these strategies are not entirely optimal, they minimize the costly mental effort needed to calculate the best route (if this is even possible) and decrease the chances of getting lost.

It is probable that, like humans, other animals come up with approximate solutions to multidestination routing problems using simple heuristics (Anderson 1983), but the empirical data are limited (reviewed in: Reynolds et al. 2013; Janson 2013). While animals often choose paths between locations that are close to optimal in terms of distance (Menzel 1973; MacDonald and Wilkie 1990; Cramer and Gallistel 1997; Bureš et al. 2006; Gibson et al. 2007), the way that they do this has not been intensively studied (but see for bumblebees: Reynolds et al. 2013). In other realms of animal behavior, the application of simple rules of thumb has been shown to explain several seemingly complex phenomena, such as the movements of fish shoals and bird flocks (Couzin et al. 2002), nest building (Karsai and Pénzes 2000), and strength asymmetry in coalition formation (Bissonnette et al. 2009). The nonhuman primate species that have been examined for their route choices have generally shown only limited ability to plan one to two steps ahead (Janson 2000, 2013). Captive vervet monkeys (*Chlorocebus pygerythrus*) have shown the greatest capacity to solve TSP‐like problems, by choosing the shortest possible route among six food sites (Cramer and Gallistel 1997). They also appeared to consider the location of at least two further goals before choosing their route, leading the authors to conclude that they planned three steps ahead (Cramer and Gallistel 1997). Yet, the success of the vervets in this study has been questioned. Janson (2013) has recently suggested that their apparent success may be explained by avoidance of the experimenter during navigation trials.

**Figure 1:**
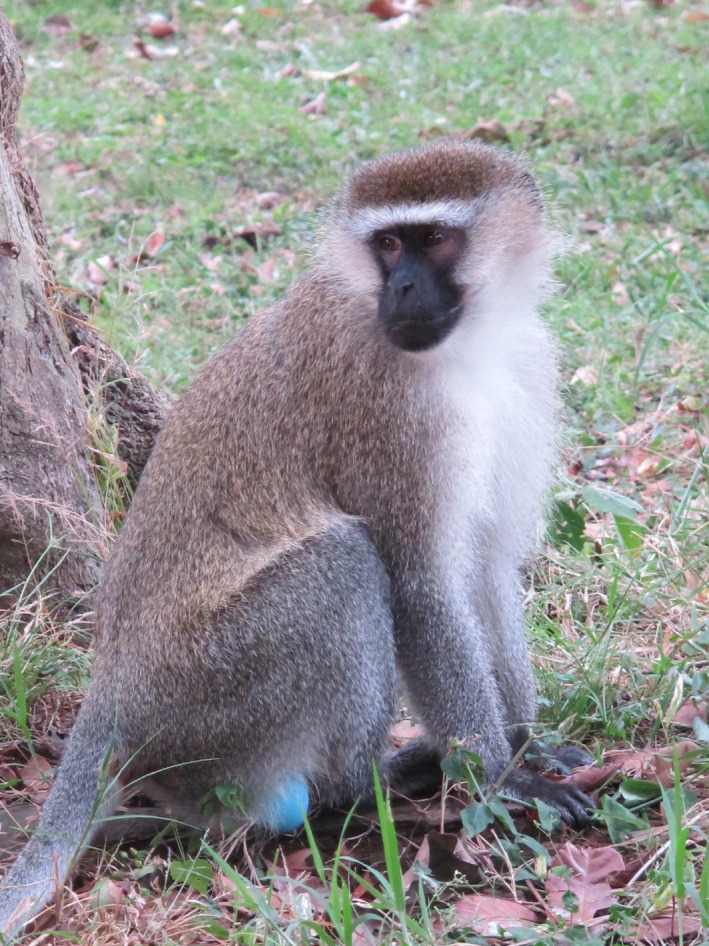
An adult male vervet monkey (*Chlorocebus pygerythrus*).

**Figure 2:**
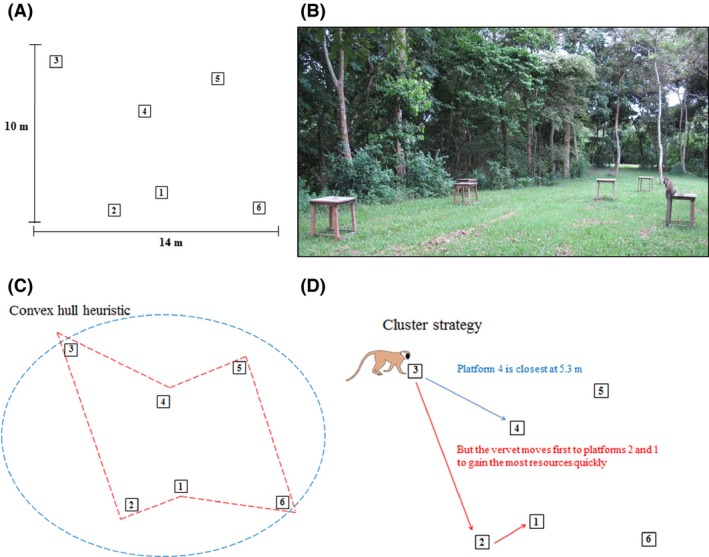
Experimental platform setup. (A) Position of experimental platforms relative to one another (B) and the platforms in the field with a study subject on Platform 5. (C) Diagram of the convex hull heuristic. To determine the order of visitation, a rubber band is conceptualized as looped around the outer targets (blue line) and pulled sequentially to the inner points (red line) in a way that stretches the band the least. (D) An example of two potential paths from Platform 3. The blue path is the shortest distance, but the red path would be taken by an individual using the cluster strategy and maximizing the number of resources obtained in the shortest amount of time.

I investigated whether a group of wild vervet monkeys (*Chlorocebus pygerythrus*, Figure 1) at Lake Nabugabo, Uganda, could solve a TSP‐like routing problem for six experimental sites or whether they used paths consistent with simple heuristics to navigate. Nonhuman primates are not usually central place foragers and do not need to return to their first destination at the end of a day, as in classical TSPs. Primate routes often mimic optimal Hamiltonian path problems (also called an open‐TSP or shortest path problem), where each destination is visited once, but an individual does not need to return to the start (Janson 2013). Path problems are at least as cognitively challenging as classical TSPs (MacGregor and Chu 2011). My goal was to examine how vervet monkeys solve a path problem by determining their use of routes consistent with three heuristics (the nearest neighbor rule (NNR), the convex hull heuristic, and a cluster strategy) in noncompetitive and competitive situations. I used feeding platforms, each baited with the same reward and placed in a clearing in the study group's home range (Fig. 2A and B) in a configuration where the shortest route from any platform was different from the route corresponding to the NNR (choose the closest site that has not been visited before) (Lihoreau et al. 2012). The shortest path could be found in a way that was not consistent with any examined heuristic from one platform (Platform 5), but in other cases, the shortest route was consistent with the convex hull heuristic (Table 1). The convex hull heuristic is conceptualized as mentally placing a rubber band around the outer points of a set of targets, then sequentially including inner points, starting with those inside targets that require the least stretching of the rubber band, until all points have been reached (Golden and Stewart 1985; Janson 2000) (Fig. 2C). This heuristic often optimally solves closed versions of TSPs but may also be used to solve open path problems if the individual begins and ends at nearby points (Chronicle et al. 2006). In the route presented to the vervets, six paths were also consistent with a cluster strategy (Table 1). Several classes of models have been developed to account for the fact that humans and other animals pay greater attention to clustered targets than to single goals within routing problems (hierarchical nearest neighbor clusters, Vickers et al. 2003; graph pyramid solution strategies, Pizlo et al. 2006; Haxhimusa et al. 2009), such that these neighborhoods of nearby sites may attract them over long distances (cluster strategy, Wiener et al. 2004; cf. Gallistel and Cramer 1996; additive gravity models, Janson 2013), and linking nearby clusters may lead to near‐optimal solutions to TSP‐like problems (nearest fragment heuristic, Ray et al. 2007). In this experiment, vervets' attraction to food sites located close together would have led to paths consistent with a cluster strategy as described by Wiener et al. (2004, 2006); where after beginning, subjects move toward large clustered targets first, to increase the number of visited sites as fast as possible, and then finish the route by moving to smaller clusters (of which there were none in this route) or to single targets (Fig. 2D).

**Table 1:** Use of paths that corresponded to a heuristic (*n *=* *276) and path distance

Shortest path (No heuristic)			Convex hull heuristic			Nearest neighbor rule			Cluster strategy		
Route	Dist. (m)	% Used	Route	Dist. (m)	% Used	Route	Dist. (m)	% Used	Route	Dist. (m)	% Used
			123,456^a^	28.4	0	124,563^b^	34.3	1.09	124,563^b^	34.3	1.09
			165,432	31.3	0						
			216,543^a^	25.5	3.26	214,563^b^	33.7	1.45	214,563^b^	33.7	1.45
			234,561	31.3	0						
			321,654	28.7	0	345,126	27.5	4.71	321,456	28.1	0.36
			345,612^a^	25.5	3.62						
			456,123^a^	28.7	1.09	451,263	36.3	0	412,653	32.4	0.72
			432,165	29.5	1.09						
561,243	26.6	1.09	543,216^a^	26.6	30.8	541,263	34.5	5.43	512,436	34.5	0.36
			561,234	29.5	0						
			612,345^a^	26.6	0	612,453^b^	27.5	3.26	612,453^b^	27.5	3.26
			654,321	28.4	0						
Total		1.09			39.9			15.94			7.24

a Route consistent with the convex hull and also the shortest path.

b Route consistent with both the nearest neighbor rule and the cluster strategy.

One group of wild vervet monkeys was used for this experiment. Some individuals would travel ahead of the group to complete trials alone, while in other cases, food competition occurred because multiple individuals were present. The goals of this experiment were to determine (1) How often single vervets found the shortest route through the food sites? (2) How much experience was needed for each individual to find the shortest path? If a large number of trials were required, this may be indicative of trial‐and‐error learning or higher cognitive processes, whereas an optimal solution found with little experience supports the use of low‐level cognitive processes like heuristics (MacGregor and Ormerod 1996; Chronicle et al. 2006). (3) How often were vervet solutions to the path problem consistent with the heuristics examined? For the entire route, the most efficient heuristic to use was the convex hull. Were paths most often consistent with the convex hull? (4) What effect did the presence of competitors have on path use? Were paths consistent with different strategies for dominant and subordinate animals in each interaction? Although the available routes for any individual depended on the actions of their competitor(s) and which food sites had already been visited, optimal strategies for each participant could be predicted. If only a portion of the food sites could be visited, high‐ranking individuals would generally do best by increasing their use of the cluster strategy because if they moved to a group of close resources, they may be able to monopolize them from subordinates. Subordinates would do best overall by increasing their use of the NNR because, when faced with competition from higher ranking individuals, they should move to the closest resource that is not being used by a competitor.

## Materials and Methods

### Study group and site

A multidestination route choice experiment was carried out with one group of wild vervet monkeys (*Chlorocebus pygerythrus*) at Lake Nabugabo, Uganda (0°22′‐12°S and 31°54′E) over 61 days from April to June 2013. The group (M group) contained 21 individuals (two adult males, seven adult females, two subadult males, one subadult female, nine juveniles, and infants) that were individually recognizable by features of the face and body. M group had a relatively predictable daily range due to their use of only two sleeping sites. Six feeding platforms (wooden tables, 0.75 m high, with a square flat top 0.75 × 0.75 m in size, Fig. 2A and B) were arranged in an experimental array between M group's sleep sites. The group passed by the platforms relatively predictably (usually twice per day) and trials were carried out on most days, whenever the monkeys ranged past the platforms (mean number of trials per day: 5.95; range: 0–40). Due to the constraints of using wild animals in experimental research, I was unable to control which individuals participated in each trial and this led to a skewed data set with unequal sample sizes for each individual (range for single trials: 2–154, range for competition trials: 1–33). The two adult males in the group dominated single trials (beta male *NM*:* n *=* *154 trials; alpha male *JK*:* n *=* *120) because of their willingness to travel away from the group.

### Study design

The arrangement of the platforms remained the same throughout the experiment so that the animals making routing decisions had prior knowledge of the distance between sites. Platforms were set in a pattern modified from Lihoreau et al. (2012), which allowed differentiation of the shortest path and routes consistent with certain heuristics (Table 1). The distances between platforms were measured precisely and flagged stakes were placed in the ground beneath to ensure that platforms were not moved between trials. The x, y coordinates of the platforms relative to one another were as follows: 1 (205.71, −257.14), 2 (111.43, −280), 3 (0.00, 0.00), 4 (165.71, −85.71), 5 (320, −45.71), and 6 (402.86, −294.29). With six sites to be visited, there were 720 possible routes (6!). Starting at each platform, the shortest path between sites could be traveled seven different ways, six of which also corresponded to the convex hull heuristic. Six routes were consistent with use of the nearest neighbor rule (NNR) or the cluster strategy, but three of these were not mutually exclusive (Table 1).

M group had been the subject of a previous foraging experiment in the same location with the same platforms the year before this study (Teichroeb and Chapman 2014), so they were quickly habituated to again receive food rewards at the site. For three days prior to data collection, platforms were baited with slices of unpeeled bananas several times per day, whenever it was noted that they were empty. During this time, the vervets learned to visit the platforms when they ranged by and they had the opportunity to become familiar with the platform arrangement. As the speed with which the vervets found the shortest route through the platforms was of interest, vervets were not trained prior to data collection and three days were considered sufficient for familiarization with platform locations. Formal trials began on April 24, 2013. During trials, each platform was baited with a small, peeled slice of banana so that individuals could grab the reward and eat it quickly before moving to the next target. Food was visible on top of platforms during trials. Platforms were not rebaited to start another trial unless all monkeys were ≥20 m away and the entire sequence could be rebaited before an individual could return. Observers recorded the identity of vervets that approached the platforms and the sequence of events for each trial including the order that sites were visited and which individual received the rewards.

The dominance relationships of the adult members of M group were established June–July 2012 based on agonistic interactions (aggression and/or submission) collected ad libitum and with focal animal samples (Teichroeb et al. 2015). Changes to these dominance rankings in 2013, due to emigrations and the maturation of individuals, were assessed ad libitum during 33 follow days (average follow: 7 h) that were conducted over the course of the experiment.

### Data analyses

In total, 361 trials were conducted by JAT and three research assistants. For 302 trials, a single forager (*n *=* *9 ind.) moved through the experimental platform setup. These were individuals that had run ahead of (or lagged behind) the rest of the group or those that could exclude other foragers from the platforms. Analyses on the route choice of single individuals were performed on complete trials, where every platform was visited once and none were revisited (*n *=* *276). A chi‐square test for homogeneity was used to determine whether each route that was used to solve the path problem was used equally. Linear mixed‐effects models were used to determine the influence of experience navigating through the route on (1) distance traveled; and (2) the number of revisits to empty platforms. Subject ID and age–sex class were included as random factors in the models to account for repeated observations on the same individuals over time. A mean percent above the optimal distance was computed for each individual that completed the route alone (Table 2).

**Table 2:** Individual differences in performance for vervets that completed the route alone

Ind.	Age–sex	Dominance rank^a^	*n* b	Mean dist. above optimal (m)	Mean % above optimal	Most common heuristic (%)
*JK*	Adult male	1	109	3.85	1.14	CH (27.5)
*NM*	Adult male	2	145	1.5	1.06	CH (49)
*MA*	Adult female	2	2	6.1	1.24	–
*TB*	Adult female	3	2	1	1.04	NNR (50), CH (50)
*LP*	Adult female	4	2	6.55	1.24	CS (50)
*TS*	Adult female	7	12	2.25	1.08	CH (41.7)
*OT*	Subadult male	3	2	4.1	1.15	CH (50)
*CL*	Subadult male	4	2	7.55	1.28	–
Overall mean			4.11	1.15		

a Within sex dominance ranking.

b Only including routes where all sites were visited, and no revisits to any platforms occurred.

CH, convex hull heuristic; CS, cluster strategy; NNR, nearest neighbor rule.

Multiple foragers were present during 59 trials involving 13 individuals. In these cases, analyses were performed on the routing decisions made by individuals (*n *=* *94 where the monkey visited at least two platforms). As there was competition for rewards during these trials, each individual only traveled through a portion of the route. On average, individual vervets visited 2.98 platforms per trial when in competition, so there was an increased incidence of paths consistent with more than one heuristic. For analysis, only trials which could be assigned unequivocally to one heuristic or another were used. Depending on the similarity between routes consistent with other heuristics, there was a higher probability of detecting some heuristics relative to others when only using a portion of the route. For instance, 12 unique routes were consistent with the convex hull heuristic, while only three were uniquely consistent with either the cluster strategy or the NNR (Table 1). So, it was more likely that the convex hull heuristic would be assigned to a certain route. In addition, while unique routes for the cluster strategy differed from the other two heuristics by the second platform choice (Table 1), the NNR and the convex hull heuristic had several routes where the first two or three platform choices were the same. Thus, the heuristic that was least likely to be detected when only a portion of the route was used was the NNR. Nonetheless, during competition trials, dominant animals visited a mean of 3.07 platforms and subordinates visited a mean of 2.89. There was no statistical difference in the number of platforms visited by dominants and subordinates (Mann–Whitney test: *n*_sub_ = 12, *n*_dom_ = 10, *Z *=* *−1.12, *P *=* *0.263), and thus, there was no difference in the probability of detecting one heuristic over the other for these categories of animals.

To examine paths consistent with certain heuristics relative to dominance status in competitive trials, the dominance rank of participants in each trial was assessed. Then, the paths consistent with each heuristic were tallied for dominant and subordinate individuals and compared with a Fisher's exact test. In addition, paths consistent with each heuristic were compared in competitive versus noncompetitive situations for individuals with sufficient trial samples sizes (*n *≥* *12) using Fisher's exact tests. For subordinates in these analyses, it was ensured that only interactions where the individual was definitely the subordinate were included. Statistics were carried out in R version 3.0.2 (R Core Team 2013) and PASW version 22.0. Tests were two‐tailed with an alpha level of 0.05 set for significance.

## Results

### Route choice for single foragers

**Figure 3:**
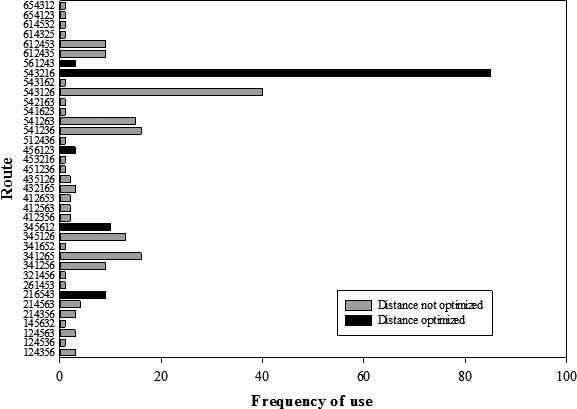
The frequency of use and optimality of the 37 different pathways that single vervets took through the route.

**Figure 4:**
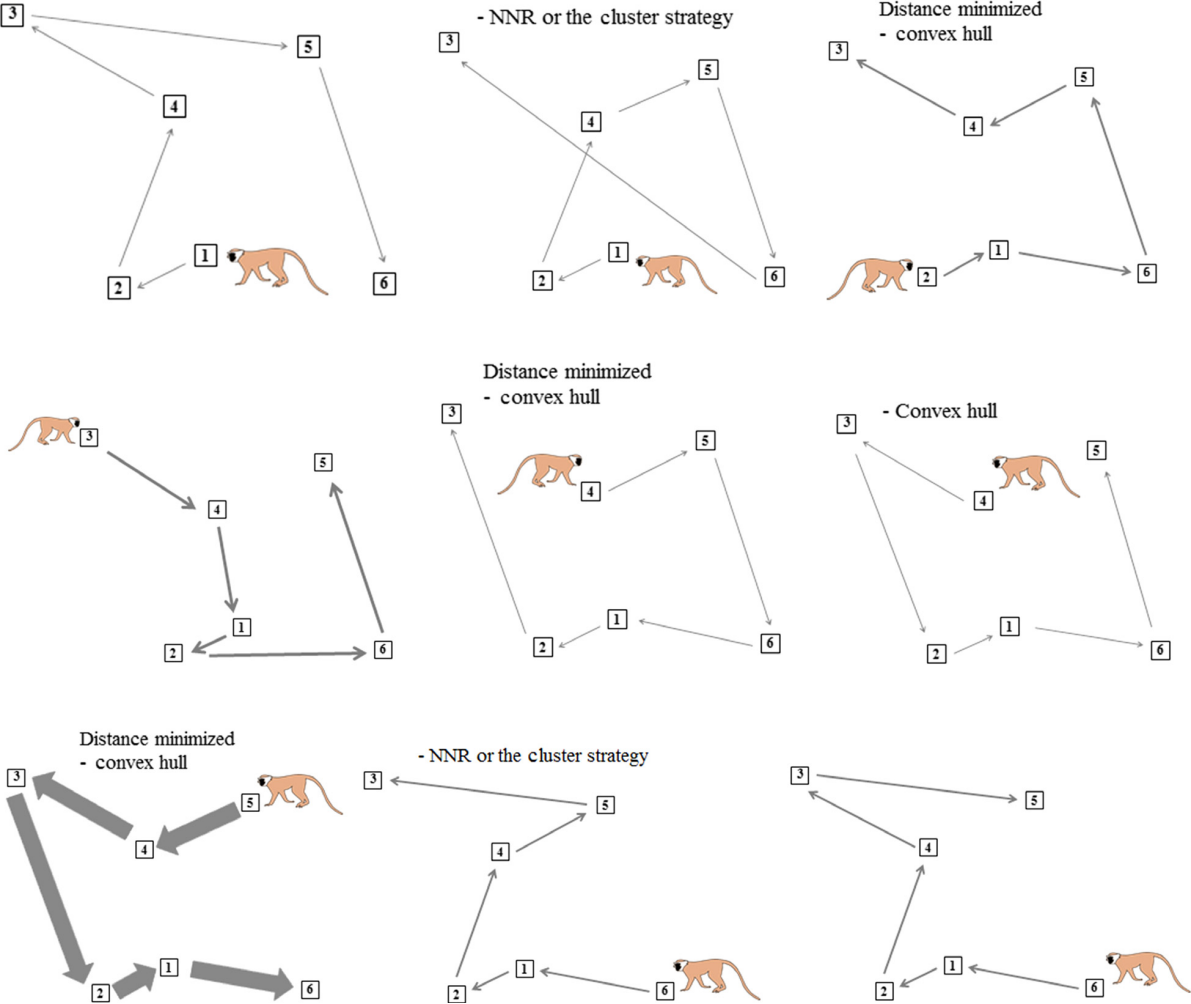
Most frequently used paths for solitary foragers from each starting position. Arrow thickness indicates the frequency of path use.

In complete trials with a single forager (*n *=* *276), vervets used 37 different routes of a possible 720. These 37 routes were not used with equal frequency (chi‐square: *χ* ^*2*^ = 1100.3, df = 36, *P *<* *0.0001; Fig. 3), and there was a tendency for vervets to approach the array from one part of their home range and begin the route at Platform 5 (59.9% of trials, *n *=* *181/302). Vervets took the overall shortest path through the platforms 39.9% of the time (*n *=* *110/276) and longer paths 60.1% of the time (166/276). The paths they took were consistent with one of three heuristics in 60.1% of trials (*n *=* *166/276) and were not consistent with any examined heuristic in 39.9% of trials (110/276). When solving the route alone, the convex hull heuristic was the most efficient (it often led to the shortest path between food sites) and paths were most often consistent with this heuristic (39.9% total) (Fig. 4). The shortest path was almost always found in a way consistent with the convex hull (38.8%). Paths were consistent with the NNR 10.1% of the time, with the cluster strategy 1.4% of the time, and with either of these heuristics an additional 11.6% of the time (Table 1).

Although, for this array, the shortest path was consistent with the convex hull heuristic in most cases, there were four sets of data that indicated that vervets used the computationally simple rule of thumb rather than calculating the shortest path between food sites. First, the speed with which each individual that found the shortest route did so. After the short period of familiarization with the route, the five individuals that found the shortest route did so after a mean of 2.2 runs through the platforms (range: 1–4) and for two of these individuals, the shortest path was found during the first recorded trial. Second, there was no effect of experience for individuals on either the distance traveled through the route (linear mixed‐effects model: df* *= 274.47, *F *=* *1.46, *P *=* *0.23) or the number of revisits to empty platforms (df* *= 287, *F *=* *1.91, *P *=* *0.17). Third, although the shortest path could be taken in a way that was not consistent with the convex hull heuristic from Platform 5, this route was only used on three occasions of 163 trials from this platform (1.8%). In contrast, route choice from this platform was optimal but consistent with the convex hull on 52% of trials. Finally, the relative lack of individual differences in successfully finding the shortest path, despite large variation in experience (*n *=* *8 ind., trial number range: 2–145, mean percent above optimal (POA) * *= 1.14%, range POA: 1.04–1.28%, Table 2) is suggestive of low‐level, automatic cognitive processes like the use of heuristics.

### Route choice during competition

**Figure 5:**
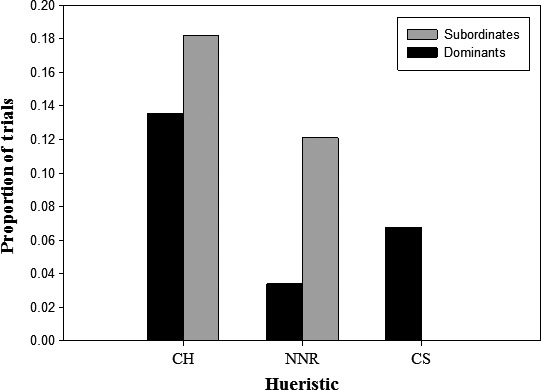
The proportion of paths consistent with each heuristic for dominants and subordinates in competitive trials (Fisher's exact test: *P *=* *0.021).

Of the 59 trials with competition, most involved two monkeys (*n *=* *53) but trials with three (*n *=* *5) and four individuals (*n *=* *1) did occur (mean* *= 2.04 ind.). One individual managed to get all of the rewards in five competition trials, while in the others (*n *=* *54), an average of 2.93 banana slices were obtained by each competitor. Dominant animals got a mean of 2.95, and subordinate animals, a mean of 2.91 banana slices, during competition trials. When in competition, there was a significant difference between the paths taken by the dominant animal in the interaction versus those taken by the subordinate. The paths of dominants were often consistent with the cluster strategy, while subordinates never used paths consistent with this heuristic. The paths taken by subordinates were more often consistent with the NNR and the convex hull heuristic compared to dominants (Fisher's exact test: *P *=* *0.021, Fig. 5).

**Figure 6:**
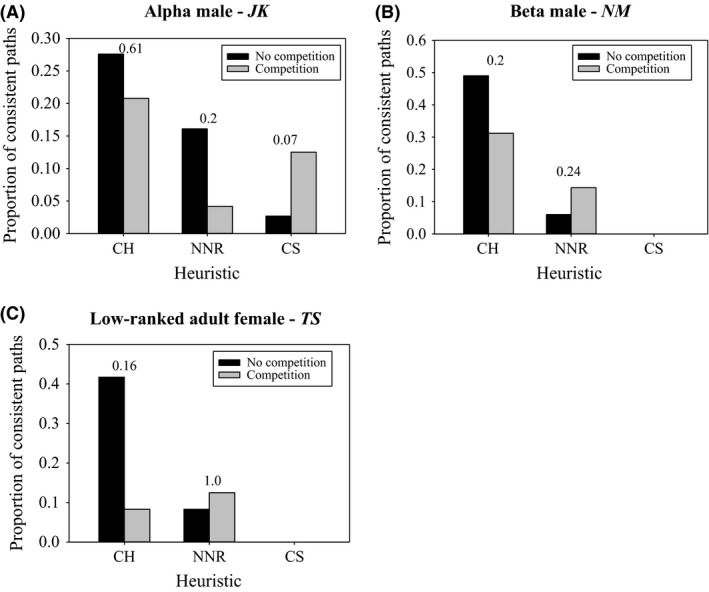
Individual paths consistent with each heuristic during competitive versus noncompetitive trials. Only individuals with *n *≥* *12 trials were examined, (A) the most dominant individual in the group, (B) the beta male, and (C) a low‐ranking adult female. *P*‐values for Fisher's exact tests appear above each set of bars.

When comparing paths consistent with each heuristic on an individual level between competitive and noncompetitive trials, the highest ranked individual in the group (alpha male *JK*) tended (Fisher's exact test: *P *=* *0.067) to have more paths consistent with the cluster strategy when competitors were present. However, there was no significant differences in his paths consistent with the convex hull heuristic (*P *=* *0.614) or the NNR (*P *=* *0.195) (Fig. 6A) between competitive and noncompetitive situations. The other two animals examined (beta male, *NM* and low‐ranked female, *TS*) never used paths consistent with the cluster strategy in either competitive or noncompetitive situations when they were the subordinate animal. They both showed a smaller proportion of paths consistent with the convex hull and a greater proportion of paths consistent with the NNR with competition but these differences were not significant (see Fig. 6B and C for *P*‐values).

## Discussion

Vervet monkey foraging is analogous to an optimal Hamiltonian path problem, and as these problems are mathematically complex, we should expect that they have evolved efficient methods of navigation that do not involve difficult calculations. The results of this study show that vervets find optimal and close‐to‐optimal paths through a multidestination route with little experience, in ways that are often consistent with simple rules of thumb. Although the shortest route in this experiment could be found in a way that was not in agreement with the convex hull heuristic, vervets very rarely used this path. Moreover, there was little difference in individual performance for monkeys completing the route (Table 2), despite substantial differences in sample sizes, which supports the use of fundamental, automatic, low‐level cognitive processes (MacGregor and Ormerod 1996; Chronicle et al. 2006).

The array used in this experiment was modified from that used by Lihoreau et al. (2012) in a study on bumblebees (*Bombus terrestris*). Like the vervets, bees were allowed to forage ad libitum in the array for a short time before data collection began. However, the monkeys appeared to optimize their routes and find the shortest path much more quickly than the bees. On average, the bees found the shortest route after 27 foraging bouts, whereas the monkeys found the shortest route, on average, after two foraging bouts, although the number of trips through the array during the familiarization process for both species was not controlled for. In agreement with the results from vervets in this study, bees were not found to use a nearest neighbor rule to optimize their travel routes, likely because it was inefficient in this particular array; bee's pathways were also more often consistent with the convex hull because it was optimal (Lihoreau et al. 2012).

The vervets in this study were also often inefficient in their route choices. Thus, contrary to the previous results of routing experiments on vervets (Cramer and Gallistel 1997), they do not seem to be different from other nonhuman primates in their abilities to find solutions to TSP‐like problems. Rather, vervets may excel at rapidly choosing the most efficient rule of thumb for a given situation, dependent on their social status. Given the extent of fast, frugal decision‐making heuristics in other realms of human and animal behavior (Gigerenzer and Todd 1999; Santos and Rosati 2015), it makes sense that these would also often be used to make spatial movement decisions. However, caution must be exercised when drawing general conclusions from these results. Given that the animals in this study were only tested on one platform array, these results could be unique to this particular route. In addition, as the shortest path in this experiment was usually consistent with the convex hull and the monkeys often used these paths, it is difficult to assess from this array whether the monkeys were calculating the shortest paths that just happened to resemble the convex hull. Future work needs to examine whether vervets apply context‐specific rules of thumb in other types of multidestination routing problems. Further, although the paths of animals were often consistent with the heuristics examined in this study, it is possible that the vervets were using different unexamined mental processes that happened to resemble the ones examined. This question deserves further scrutiny and experiments could be set up to explicitly test the use of additional heuristics, such as two‐step and three‐step look ahead rules, which vervets have been suggested to use (Cramer and Gallistel 1997). The second most commonly used route in this experiment was not consistent with any of the heuristics examined, raising the possibility that other decision rules may have been used.

Humans use heuristics when solving TSP‐like problems and the ways that we apply these rules have been widely analyzed (MacGregor and Chu 2011), although people are usually asked to solve routing problems using a pen and paper or on a computer screen rather than physically moving through space. It is noteworthy that vervets in this study appear to be using some of the same heuristics that are often applied by humans – the convex hull (MacGregor and Ormerod 1996; Ormerod and Chronicle 1999; MacGregor et al. 2000), the nearest neighbor rule (Gärling 1989; Hirtle and Gärling 1992), and the cluster strategy (Wiener et al. 2004, 2006). There are two main hypotheses that can be put forward to explain these similarities. One possibility is that spatial movement strategies are conserved in the primate lineage. As the line that lead to Old World monkeys is suggested to have diverged from the ancestral line leading to humans about 23 million years ago (Raaum et al. 2005), these results suggest a relatively ancient evolutionary origin for some human navigation rules. This is in‐line with recent evidence showing that several other psychological mechanisms (e.g. the framing effect, peak‐end effect, and temporal and effort discounting.) appear to be conserved in our lineage because humans share them with nonhuman primates (Santos and Rosati 2015). In actuality, although much more research needs to be carried out, work with other animal species suggests that the nearest neighbor rule (bumblebees: Ohashi et al. 2007; rats: Blaser and Ginchansky 2012), the convex hull heuristic (bumblebees: Lihoreau et al. 2012), and the cluster strategy (vervets: Gallistel and Cramer 1996; Cramer and Gallistel 1997) may be used more widely to solve multidestination routing problems. Thus, certain spatial movement heuristics may be part of a “universal mental toolkit” that all animals share (Hauser 2000). Given the taxonomic breadth of animals that have shown support for some of these heuristics, it is equally plausible that these heuristics arose independently in several animal lineages through a process of convergent evolution because they are relatively optimal at solving multidestination routing problems given time and knowledge constraints.

This research also demonstrated that routing decisions for nonhuman primates are altered by the presence of others. For most socially foraging species, it is difficult to know what movement decisions would have been made, had animals been foraging alone, because group mates are always present. The tendency of vervets in this study to travel ahead of the group and complete experiments alone, before rejoining the group (to again go through the route), provided a unique opportunity to examine decision‐making in a solitary and a social foraging situation. Relative to nonsocial environments, social ones are more complex, unpredictable, and challenging (Humphrey 1976; Byrne and Whiten 1988) and this intractableness calls for robust, rapid, optimization strategies, such as heuristics, that can be used before competitors have the opportunity to react (Hurley 2005; Hertwig and Herzog 2009). When alone, an individual must make decisions based on physical space but when with others, strategies also need to be used to beat out food competitors. Thus, the optimal behavior of an individual is going to differ depending on the situation. Without competitors, vervet paths were often consistent with the convex hull heuristic, which in many cases allowed them to travel the shortest distance while getting every reward (solving the path problem). When others were present, individual decisions seemed to be made much more quickly and food sites needed to be prioritized based on their distance, nearness to other food sites, and whether they had already been visited. As predicted, there was support for dominant animals increasing their use of paths consistent with a cluster strategy when competitors were present and subordinates increasing their use of paths consistent with the NNR. For subordinates that are unlikely to get any resources unless they rush to the closest one, it is possible that they discount future foraging opportunities when with a dominant and thus apply a simple one‐step look ahead rule (the NNR). Using a cluster strategy, dominant vervets were increasing their likelihood of getting more than one resource, so it is possible that they were applying a two‐step (or more) look ahead rule, a possibility that needs further examination. The dispersion of food resources is another important variable when assessing the usefulness of a cluster strategy to dominant animals; their abilities to monopolize the resources within a cluster likely decrease when grouped food items are too far apart.

Finally, this study showed that vervet monkeys may have the ability to choose between optimal spatial movement heuristics depending on their dominance rank. Relative social status often changes in certain situations and during a primates' lifetime (especially for males), so it is an interesting question whether evolution prepared vervets with an arsenal of heuristics that could be used depending on their current dominance rank relative to competitors, or alternatively, whether individuals can learn new rules of thumb based on experience? Relatively, little research has been carried out to determine the extent of heuristic use in animal navigation, and this is an area ripe for greater inquiry.

## Conflict of Interest

None declared.
